# Association between Thyroid Dysfunction and Intensive Care Unit-Acquired Weakness: A Case-Control Study

**DOI:** 10.1155/2021/8889036

**Published:** 2021-09-28

**Authors:** Tarek Samir Shabana, Sherif George Anis, DiaaElDein Mahmoud Ibrahim

**Affiliations:** Faculty of Medicine, Ain Shams University, Cairo, Egypt

## Abstract

**Background:**

Thyroid disorders may decrease the threshold for developing myopathy. Nonthyroidal illness syndrome (NTIS) is a common form of thyroid dysfunction in critically ill patients who are prone to the development of intensive care unit-acquired weakness (ICUAW). We therefore tested the hypothesis that patients with abnormalities in thyroid function are at a higher risk of developing ICUAW.

**Methods:**

We assessed blood samples from patients admitted to the ICU for ≥7 days for thyroid functions. Patients were classified into 4 categories (euthyroid, hyperthyroid, hypothyroid, and NTIS). Patients were then evaluated daily for ICUAW development. Patients with ICUAW were considered as cases, whereas patients who did not develop ICUAW served as controls. We compared demographic and clinical variables, such as APACHE II score; length of ICU stay; free T3 (FT3), free T4, and thyroid-stimulating hormone levels; incidence of the four categories of thyroid function; and other risk factors for ICUAW. Logistic regression was used to determine independent risk factors for ICUAW.

**Results:**

This case-control study included 114 patients: 57 cases (ICUAW) and 57 controls. FT3 levels were significantly lower in the cases (2.13 ± 0.96 mU/L) than in controls (2.69 ± 1.07 mU/L; *P*=0.004). There were no significant differences between cases and controls regarding the incidence of all categories of thyroid function. In univariate analysis, five independent variables had *P* < 0.25 (sepsis, vasopressors, mechanical ventilation duration, NTIS, and FT3 levels). Among these variables, multiple regression showed that only FT3 level (CI = 0.157–0.82, *P*=0.015) was an independent risk factor.

**Conclusion:**

The study revealed an inverse association between ICUAW incidence and FT3 levels.

## 1. Introduction

Intensive care unit- (ICU-) acquired weakness (ICUAW) refers to muscle weakness that develops as a result of critical illness. It affects more than 50% of patients in the ICU and is related to many problems such as difficult weaning from mechanical ventilation (MV), prolonged hospital stay, and increased mortality risk [1]. Neurophysiological studies have further classified ICUAW into three categories: critical illness myopathy (CIM), critical illness polyneuropathy, and critical illness neuromyopathy [2].

Identification of risk factors for ICUAW development is the first step in the prevention and management of this disorder. Sepsis, prolonged ICU stay, prolonged MV, hyperglycaemia, and high-dose steroid therapy are reported as the most important risk factors [3].

Thyroid disorders are also associated with neuromuscular abnormalities and may therefore decrease the threshold for developing any type of myopathy [4]. Moreover, patients with critical illness, who are typically prone to ICUAW development, have changes in their thyroid biochemistry, namely, low free T3 (FT3) levels (with or without low free T4 (FT4) levels), and normal levels of the thyroid-stimulating hormone (TSH). These changes are collectively known as nonthyroidal illness syndrome (NTIS; previously, low T3 syndrome and euthyroid sick syndrome) which is the most common form of thyroid dysfunction in the ICU [5].

Thus far, no study has investigated the direct relationship between thyroid dysfunction and ICUAW development. Therefore, in the present study, we aimed to test the hypothesis that patients with abnormalities in thyroid function are at a higher risk of developing ICUAW.

## 2. Materials and Methods

This case-control study was conducted in 114 critically ill patients admitted to Ain Shams University Intensive Care Units. Institutional ethical clearance (FMASU R10/2020) and informed consent from the patients' first-degree relative or guardian were obtained. The study was registered in ClinicalTrials.gov (NCT04313101).

### 2.1. Enrollment

Blood samples were collected from patients admitted to the ICU for ≥7 days (as these patients are more likely to develop ICUAW) to assess their thyroid function. These patients were then evaluated daily for the presence of ICUAW. Fifty-seven consecutive patients who were diagnosed with ICUAW were included in the study as cases. Another 57 patients who did not develop ICUAW during their ICU stay were included as controls.

### 2.2. Exclusion Criteria

Patients with cerebrovascular accidents, neuromuscular disorders, spinal cord or head injuries, tumors in the central nervous system, secondary thyroid disorders, liver cell failure, and end-stage renal disease were excluded from the study. Patients receiving thyroid replacement or using antithyroid drugs or other drugs known to affect thyroid function (e.g., amiodarone, lithium, oestrogen, dopamine agonists, and somatostatin analogues) were also excluded from the study because these drugs may alter their thyroid biochemistry. Brain imaging was performed only for patients with a history or clinical examination findings suggestive of a recent neurological disorder.

### 2.3. Diagnosis of ICUAW

The diagnosis of ICUAW was made depending on clinical criteria for ICUAW, and the findings were confirmed by nerve conduction studies.

### 2.4. Clinical Assessment

Neurological examination was performed once daily for patients who were admitted to the ICU for ≥7 days. Muscle power was assessed bilaterally for each of the following movements: shoulder abduction, elbow flexion, wrist extension, hip flexion, knee extension, and ankle dorsiflexion. Muscle tone was evaluated in uncooperative patients or patients with disturbed sensoria.

The Medical Research Council (MRC) muscle power scale was calculated in cooperative patients to determine their score out of 60 (Table 1).

The clinical criteria for ICUAW [7] were as follows:

1. Weakness developing after the onset of critical illness
2. Generalised (proximal and distal), symmetrical, and flaccid weakness; cranial nerves spared
3. MRC sum score <48
4. Mechanical ventilator dependence
5. Cause of weakness not related directly to the underlying critical illness

The diagnosis of ICUAW was made depending on the presence of criteria (1), (2), and (5) in addition to (3) or (4).

Ventilator dependence was considered in cases with failure in at least three trials of weaning or weaning process persisting for more than 7 days after the first spontaneous breathing trial [8].

### 2.5. Nerve Conduction Studies

Unilateral bedside nerve conduction tests of the sural and peroneal nerves were performed in patients who fulfilled the clinical criteria for ICUAW. The diagnosis of ICUAW was confirmed by the presence of reduced compound motor action potential (CMAP) amplitudes. CMAP represents the summated action potentials of all the stimulated motor endplates [9, 10]. No further attempts were made to differentiate different subtypes of ICUAW using electromyography, direct muscle stimulation, or muscle biopsy.

### 2.6. Thyroid Function Testing

Venous blood samples were withdrawn from all patients admitted to the ICU for ≥7 days to measure the levels of thyroid hormones (FT3: triiodothyronine, FT4: thyroxine, and TSH) using enzyme-linked immunosorbent assay (ELISA) kits. Reference ranges were as follows: TSH, 0.4–4 mU/L; FT3, 2–4.4 mU/L; and FT4, 0.8–1.9 mU/L. Patients were then categorised into one of the following categories:

- Euthyroid (normal levels of TSH, FT3, and FT4)
- Hyperthyroid (low TSH levels), either overt (increased FT4 ± FT3 levels) or subclinical (normal levels of FT4 and FT3)
- Hypothyroid (elevated TSH levels), either overt (low FT4 ± FT3 levels) or subclinical (normal FT3 and FT4)
- NTIS: normal or low TSH levels in addition to low FT3 ± low FT4 levels

This categorisation of thyroid dysfunction was based on data published in a previous study [11], which also required both FT3 and FT4 levels to be either low or high to diagnose hypothyroidism or hyperthyroidism, respectively. However, in the current categorisation, abnormalities in FT4 levels alone together with TSH abnormalities were accepted for the diagnosis of both hypo- and hyperthyroidism according to the American Thyroid Association [12].

Other risk factors for ICUAW were identified as follows: (1) sepsis, defined as organ dysfunction evident by an increase in the Sequential Organ Failure Assessment score of 2 or more points from baseline in the presence of infection [13]; (2) treatment with corticosteroids (more than 10 g of hydrocortisone or its equivalents in less than 2 weeks), aminoglycosides, or neuromuscular blockers (NMBs, of any type) for 3 days or more either continuously or intermittently) [7]; (3) hyperglycaemia: mean blood glucose levels (from the time of ICU admission to enrollment) >180 mg/dL [14]; (4) need for vasopressors; (5) MV duration; and (6) protein-calorie malnutrition.

Comparisons were performed between groups with regard to demographics; APACHE II score; length of ICU stay before the diagnosis of ICUAW (cases) or end of ICU stay (controls); FT3, FT4, and TSH levels; and the incidence of the four categories of thyroid function as well as other risk factors for ICUAW. Logistic regression was used to determine independent risk factors for ICUAW.

### 2.7. Statistical Analysis

SPSS Statistics (V.210.0, IBM Corp., USA, 2015) was used for data analysis. Categorical data were analysed using the chi-square test. Numerical data were analysed using Student's *t*-test. Statistical significance was set as *P* < 0.05. Data were expressed as mean (standard deviation, SD) for quantitative parametric analysis, and independent variables were assessed using univariate binary logistic regression. Variables with *P* value >0.25 in univariate analysis were included in a multivariate model.

### 2.8. Sample Size Calculation

As there were no previously published data regarding the relationship between thyroid function and ICUAW development, sample size was calculated assuming the incidence of abnormalities in thyroid function in patients with ICUAW to be 50% compared with that of 20% in controls at an alpha error of 0.05 and a study power of 90%. Accordingly, a sample of at least 114 patients was needed (57 cases and 57 controls).

## 3. Results

There were no significant differences between cases (ICUAW) and controls with regard to age, sex, APACHE II scores, length of ICU stay, and BMI (*P*=0.91, 0.34, 0.77, 0.55, and 0.85, respectively) (Table 2).

FT3 levels were significantly lower in cases (ICUAW) compared to controls (*P*=0.004). FT4 and TSH levels were comparable between the two groups (*P*=0.66 and 0.21, respectively) (Table 3).

There were no significant differences between cases (ICUAW) and controls with regard to the incidence of euthyroidism, hyperthyroidism, hypothyroidism, and NTIS (*P*=0.05, 0.2, 0.75, and 0.13, respectively) (Table 4).

The duration of MV was significantly longer in patients with ICUAW than in control subjects (*P* < 0.01). The need for vasopressors was also significantly higher in patients with ICUAW than in control subjects (*P*=0.01). There were no significant differences between cases and controls with regard to the incidence of other risk factors for ICUAW: hyperglycaemia, use of NMBs, aminoglycosides, sepsis, steroids, and protein-calorie malnutrition (*P*=0.54, 1, 0.62, 0.18, 0.7, and 0.3, respectively) (Table 5).

Twenty-one (56.75%) cases with sepsis required vasopressors compared with 9 (30%) control subjects with sepsis (*P*=0.02). As for NTIS, 23 (71.87%) cases had sepsis compared with 19 (79.16%) control subjects (*P*=0.53), and 20 (35.08%) cases were mechanically ventilated compared to 4 (7.01%) control subjects (*P* < 0.001); 19 (33.33%) cases required vasopressors compared with 7 (12.28%) control subjects (*P*=0.02), and 15 cases (46.87%) were treated with steroids compared to 12 (50%) control subjects (*P*=0.81).

In the univariate analysis, five independent variables had *P* < 0.25 (sepsis, use of vasopressors, MV duration, NTIS, and FT3 levels) (Table 6). These variables were included in a multiple regression model in the multivariate analysis in which only FT3 level was an independent risk factor (CI = 0.157–0.82, *P*=0.015) (Table 7).

## 4. Discussion

The current study revealed that ICUAW development was associated with lower FT3 levels.

In 2004, Riggs et al. reported the case of a 51-year-old woman who developed CIM after prolonged intubation for chronic obstructive pulmonary disease (COPD) exacerbation. The patient received steroids, antibiotics, and NMBs. She was found to have hyperthyroidism. Her weakness gradually improved within 1 month of treatment with iodine and methimazole. Apart from this case report, no previous study has assessed the direct relationship between ICUAW development and thyroid dysfunction [15].

The hypothesis in the current study was based on the fact that the prevalence of muscle weakness is high in patients with thyroid dysfunction [16, 17]. The pathophysiological mechanisms by which thyroid disorders predispose to muscle weakness are diverse. While hyperthyroidism is associated with accelerated protein catabolism and release of calcium from the sarcoplasmic reticulum, hypothyroidism results in impaired mitochondrial energy production. Thyroid disorders may also play a role in the development of sarcopenia, a component of the pathophysiology of ICUAW, especially in the elderly [18].

However, in the current study, categorisation of patients was based merely on thyroid biochemistry. For this reason, patients with known thyroid abnormalities receiving thyroid replacement and those receiving antithyroid medications were excluded at baseline as these drugs may alter their thyroid biochemistry. Eventually, NTIS and undiagnosed hyper- and hypothyroidism (overt and clinical) were the only expected thyroid abnormalities after testing for thyroid function. Moreover, the relatively small sample size resulted in a small percentage of patients with hyper- and hypothyroidism, which are less common than NTIS in ICU patients, after categorisation.

Difficulty in weaning from MV is a clinical criterion for diagnosing ICUAW. Previous studies have focused on thyroid dysfunction as a risk factor for difficulty in weaning from MV. These studies have shown conflicting results. A study carried out by Datta and Scalise [19] reported hypothyroidism as an uncommon cause of difficulty in weaning from MV. These results were supported by a study conducted by Pandya et al. [20]. According to Yasar et al. [21], NTIS was an independent predictor of prolonged weaning in patients with COPD admitted to the ICU. Bello et al. [22] also reported NTIS as a risk factor for prolonged MV.

In the current study, FT3 levels were significantly lower in patients with ICUAW than in controls. Low FT3 levels have been previously linked to increased mortality, poor outcomes, and the need for MV in the ICU [23–26]. FT3 levels correlate with adverse outcome in patients with coronary artery disease and in patients with respiratory failure [27, 28].

Low FT3 is a feature of NTIS, a condition associated with altered metabolism of thyroid hormones peripherally as well as in the hypothalamic-pituitary axis function in critically ill patients. Low FT3 levels may be linked to ICUAW development through several factors. ICUAW is commonly found in critically ill patients. Low FT3 levels reflect severe illness and advanced disease. Although there were no significant differences in the incidence of NTIS between cases and controls in the current study, the incidence of sepsis and the need for vasopressors were higher among cases with NTIS than in control subjects with the same condition.

Moreover, there is an overlap in the pathophysiology of both NTIS and ICUAW. Inflammatory cytokines (e.g., interleukin-6, tumour necrosis factor, and interferon-*α*) which are related to thyroid function abnormalities are also involved in the pathogenesis of ICUAW [29]. Sepsis and corticosteroids, which are confounders for ICUAW, also affect the concentration and binding ability of thyroxin-binding globulin [30].

Interestingly, low FT3 levels have also been found to correlate inversely with markers of muscle breakdown reflecting an association between low FT3 levels and hypercatabolism associated with critical illness [31].

Skeletal muscle biopsies from prolonged critically ill patients, who are typically prone to ICUAW, revealed overexpression of both monocarboxylate transporter MCT-8 (an active thyroid hormone transporter) and type 2 deiodinase responsible for the conversion of T4 to T3. This may be a mechanism by which the body responds to decreased FT3 levels, aiming to increase tissue availability [32].

Apart from low FT3 levels, prolonged MV remains an important risk factor for ICUAW development and has been found to have a predictive value. Prolonged MV may result in diaphragmatic muscle weakness, leading to difficulty in weaning and a longer duration of MV ending in a vicious circle. According to Mirzakhani et al., the incidence of ICUAW in patients on MV support for more than 10 days reached 67% [33]. In the current study, the duration of MV was significantly longer in patients with ICUAW than that in control subjects. Published studies have also identified sepsis, severity of illness scores, and multiorgan failure as other nonmodifiable risk factors for ICUAW. In patients with systemic inflammatory response syndrome and sepsis, proinflammatory mediators result in muscle damage and axonal degeneration by altering signalling pathways [34]. In the current study, although the incidence of sepsis was comparable between patients with ICUAW and control subjects, the need for vasopressors was significantly higher among ICUAW patients with sepsis, indicating a higher incidence of septic shock. Both MV duration and presence of sepsis were significantly associated with ICUAW development in a meta-analysis conducted by Yang et al. in 2018 [3]. Hyperglycaemia has also been listed as a probable risk factor for ICUAW development, with intense insulin therapy in the ICU as a possible prevention strategy [35]. Previous studies have focused on the use of drugs such as aminoglycosides, NMBs, and high-dose steroids as modifiable risk factors for ICUAW incidence, although with conflicting results [36].

### 4.1. Limitations

A limitation of this study was that muscle biopsy was not performed. Biopsy is the standard of care approach for diagnosing ICUAW and differentiating it from pure thyroid myopathy.

Another important limitation is the small sample size, which resulted in small proportions of patients in each category of thyroid function after categorisation.

Further multicentre studies with larger sample sizes are needed to support the findings of the current study and to determine whether FT3 levels can be used to predict the incidence of ICUAW.

## 5. Conclusion

ICUAW development is inversely associated with FT3 levels.

## Figures and Tables

**Table 1:** MRC muscle power scale [6].

Strength	Score
No contraction	0
Flicker or trace of contraction	1
Active movement with gravity eliminated	2
Active movement against gravity	3
Active movement against gravity and resistance	4
Normal power	5

MRC: Medical Research Council.

**Table 2:** Patient demographics and length of ICU stay.

	ICUAW cases	Controls	*P* value
Age (years)	58.94 (9.95)	59.14 (9.78)	0.91
Sex (M/F)	25/32	30/27	0.34
APACHE II score	24.03 (3.63)	23.84 (3.49)	0.77
LOS (days)	21.92 (7.58)	22.82 (8.74)	0.55
BMI (kg/m^2^)	74.03 (17.99)	74.66 (18.11)	0.85

Data are presented as mean (SD). APACHE: Acute Physiology and Chronic Health Evaluation; BMI: body mass index; ICUAW: intensive care unit-acquired weakness; ICU: intensive care unit; LOS: length of stay.

**Table 3:** Thyroid function tests' results (mU/L).

Thyroid hormones	ICUAW cases	Controls	*P* value
FT3	2.13 (0.96)	2.69 (1.07)	0.004^*∗*^
FT4	1.28 (0.37)	1.25 (0.36)	0.66
TSH	2.84 (1.98)	2.34 (2.24)	0.21

Data are presented as mean (SD). ICUAW: intensive care unit-acquired weakness; FT3: free triiodothyronine; FT4: free thyroxine; TSH: thyroid-stimulating hormone. ^*∗*^*P* < 0.05.

**Table 4:** Incidence of thyroid dysfunction in both groups.

Thyroid disorder	ICUAW cases	Controls	*P* value
Euthyroid	16 (28.07%)	26 (45.61%)	0.05
Hyperthyroid	3 (5.26%)	2 (3.5%)	0.2
Hypothyroid	6 (10.52%)	5 (8.77%)	0.75
NTIS	32 (56.14%)	24 (42.1%)	0.13

Data are presented as number (%) of patients. ICUAW: intensive care unit-acquired weakness; NTIS: nonthyroidal illness syndrome.

**Table 5:** Other risk factors for ICUAW.

Risk factor	ICUAW cases	Controls	*P* value
Hyperglycaemia	17 (29.82%)	20 (35.08%)	0.54
NMBs	1 (1.75%)	1 (1.75%)	1
Aminoglycosides	11 9 (19.29%)	9 (15.78%)	0.62
Sepsis	37 (64.91%)	30 (52.63%)	0.18
Steroids	26 (45.61%)	24 (42.1%)	0.7
Vasopressors	28 (49.12%)	15 (0.26%)	0.01^*∗*^
MV duration (days)	6.4 (8.56%)	0.89 (2.53%)	<0.001^*∗*^
Protein-calorie malnutrition	11 (19.29%)	7 (12.28%)	0.3

Data are presented as number (%) of patients. Data of MV duration are presented as mean (SD). NMBs: neuromuscular blockers; MV: mechanical ventilation; ICUAW: intensive care unit-acquired weakness. ^*∗*^*P* < 0.05.

**Table 6:** Univariate analysis of ICUAW predictors.

Predictor	Exp (*B*)	95% CI	*P* value
APACHE II score	1.015	0.916–1.125	0.77
Euthyroidism	0.925	0.427–2.006	0.84
Hyperthyroidism	10.655	0.105–4.073	0.65
Hypothyroidism	0.817	0.235–2.847	0.75
NTIS	0.61	0.291–1.279	0.19
FT3 level	0.586	0.403–0.852	<0.001^*∗*^
Hyperglycaemia	1.385	0.626–3.063	0.54
NMBs	1	0.061–16.386	1
Aminoglycosides	0.683	0.252–1.848	0.45
Sepsis	0.601	0.283–1.275	0.18
Steroids	0.867	0.414–1.818	0.7
MV duration	1.236	1.097–1.393	<0.001^*∗*^
Vasopressors	0.337	0.152–0.747	0.007^*∗*^
Protein-calorie malnutrition	0.585	0.209–1.638	0.3

APACHE: Acute Physiology and Chronic Health Evaluation; NTIS: nonthyroidal illness syndrome; NMBs: neuromuscular blockers; MV: mechanical ventilation; FT3: free T3; CI: confidence interval; ICUAW: intensive care unit-acquired weakness. ^*∗*^*P* < 0.05.

**Table 7:** Multivariate analysis of ICUAW predictors.

Predictor	Wald	Exp (*B*)	95% CI	*P* value
Vasopressors	1.92	0.515	0.201–1.317	0.16
NTIS	3.281	4.334	0.887–21.181	0.07
Sepsis	0.83	1.568	0.596–4.128	0.36
FT3 level	5.917	0.359	0.157–0.82	0.015^*∗*^
MV duration	8.6	1.153	0.964–1.377	0.003^*∗*^

NTIS: nonthyroid illness syndrome; MV: mechanical ventilation; FT3: free T3; CI: confidence interval; ICUAW: intensive care unit-acquired weakness. ^*∗*^*P* < 0.05.

## Data Availability

The authors confirm that the data supporting the findings of this study are available within the article. Further data may be provided by the corresponding author upon request.
